# A Sweeter Pill to Swallow: A Review of Honey Bees and Honey as a Source of Probiotic and Prebiotic Products

**DOI:** 10.3390/foods11142102

**Published:** 2022-07-15

**Authors:** Suraiami Mustar, Nurliayana Ibrahim

**Affiliations:** Nutrition Unit, Nutrition, Metabolism and Cardiovascular Research Centre, Institute for Medical Research, National Institutes of Health, Ministry of Health Malaysia, Block C7, Level 3, No. 1, Jalan Setia Murni U13/52, Seksyen U13, Setia Alam, Shah Alam 40170, Malaysia; liayana.psh@moh.gov.my

**Keywords:** probiotic, prebiotic, oligosaccharides, lactic acid bacteria, honey bee, honey

## Abstract

Honey bees and honey, have been the subject of study for decades due to their importance in improving health. At times, some of the probiotics may be transferred to the honey stored in the honeycomb. Consumers may benefit from consuming live-probiotics honey, which can aid in suppressing the reproduction of pathogens in their digestive system. Prebiotics, on the other hand, are mainly carbohydrates that promote the growth of native microflora probiotics in the digestive tract to maintain a healthy environment and improve the gut performance of the host. Therefore, this narrative review aims to present and analyze ten years’ worth of information on the probiotic and prebiotic potential of honey bees and honey since not many review articles were found discussing this topic. Results showed that not many studies have been performed on the probiotic and prebiotic aspects of honey bees and honey. If further research is conducted, isolated probiotics from the bee’s gut combined with honey’s prebiotic properties can be manipulated as potential sources of probiotics and prebiotics for human and animal benefits since they appear to be interrelated and function in symbiosis.

## 1. Introduction

Honey bees come from the family of Apidae and the genus *Apis* [1]. *A. dorsata*, *A. mellifera*, *A. cerana*, *A. laboriosa*, *A. florea*, *A. andreniformis*, *A. koschevnikovi*, and *A. nigrocincta* are eight known species that can be found around the world [2]. Honey bees are significant pollinators for cultivating crops for food production, ensuring the continuity of almost all life in this world [3]. The honey bee’s gut contains many microorganisms as its normal microbiota. Most are probiotics, made up of lactic acid bacteria (LAB) and *Bifidobacterium*, which are widely distributed in their digestive tract system [4]. Probiotics were first described in 2013 by the International Scientific Association for Probiotics and Prebiotics (ISAPP) [5] as “live microorganisms that, when administered in adequate amounts, confer a health benefit on the host”. The scientific definition has been extensively applied around the globe. Probiotics enhance intestinal health and increase immune reaction by producing biological antimicrobial substances that can inhibit pathogens which caused digestive system imbalances in humans and animals. Probiotics have been shown in several studies to minimize the occurrence of diarrhea, allergy, lactose intolerance, cancer, and lower serum cholesterol [6]. The normal microbiota species of bees are influenced by environmental factors such as bee foraging activity on various types of flowers, contact with older bees, and maternal inheritance [4,7,8]. Various industrial sectors, such as the food, feed, and pharmaceutical companies, could benefit from the honey bee probiotic microorganisms [9].

Many products produced by honey bees are useful to humans, including honey, [10,11] which is the most important and widely consumed bee product worldwide. Honey, a “natural sweet substance produced by *Apis mellifera* L. bees from the nectar of plants, secretions of living parts of plants, or excretions of plant-sucking insects on the living parts of plants, which the bees collect, transform by combining with specific substances of their own, deposit, dehydrate, store and leave in the honeycomb to ripen and mature” [12], comes in two varieties namely: blossom/nectar honey and honeydew honey. Blossom honey is made from flowering plant nectar, whereas honeydew honey is manufactured from honeydew collected from various parts of a plant or other sap-producing plants and insects [13]. Honey can be divided into two categories: unifloral (monofloral) and polyfloral (multifloral). Unifloral honey is made primarily from one type of plant nectar and is identified through pollen analysis, which reveals dominant pollen from a single plant species. Polyfloral honey does not have dominant pollen from one plant species but has a mixture of pollen from several plants [14]. Due to its refined, one-of-a-kind, and distinct flavor, unifloral honey typically commands a higher market price than polyfloral honey. The premium quality of unifloral honey mostly depends on the exclusive geographical area or the special plant species, for example, the Manuka honey from New Zealand [15]. Honey may contain probiotics that have been transmitted from the guts of honey bees during the process of making honey and may remain alive for a certain period [16]. Thus, both honey bees and honey may provide potential probiotics for future use. The health benefits of honey concerning its probiotic bacteria are that the probiotics will help to revitalize and strengthen the immune system of the host against harmful environmental factors and pathogens, aid in digestion, detoxify harmful substances and provide essential nutrients [6].

Honey is mostly made up of sugars or carbohydrates such as fructose (32–44%), glucose (23–38%), and some other complex sugars (5–15%) including sucrose, maltose, lactose, raffinose, trehalose, erlose, gentiobiose, turanose, panose, melezitose, and kojibiose amongst others [17]. Besides carbohydrates, the quality and health advantages of honey are also ascribed to the various components it possesses, such as protein, organic acids, amino acids, vitamins, minerals, enzymes, and polyphenols [18]. Different varieties of honey may vary in their content due to the different sources derived, such as geographical area, botanical origin, and bee species [17]. Blossom or nectar honey can be distinguished from honeydew honey by analyzing its carbohydrate concentration. Blossom honey contains higher concentrations of monosaccharides but is lower in trisaccharides (mainly melezitose, erlose, raffinose, and maltotriose) and other oligosaccharides compared to honeydew honey [19]. The honey’s prebiotic properties are known to come from its indestructible carbohydrates that cannot be fermented by digestive enzymes in humans and are not taken up in the upper intestinal tract system. They are capable of improving and enhancing health in general and intestinal health in particular by stimulating the development and promoting metabolic activity of the typical residents of the colon [20]. Honey’s prebiotic qualities can help probiotic microorganisms to flourish by supplying adequate nutrients. An increased number of probiotics may help to alleviate the total surface area for nutrient absorption, thus improving the health of the digestive system and enhancing resistance to pathogen infections [21]. These findings have sparked some ideas for conducting studies for further research on the natural microbiota of the bees’ gut with probiotic properties as a disease defense mechanism to be used as prophylaxis to treat not only bees themselves but also other animals and humans [6].

**Figure 1:**
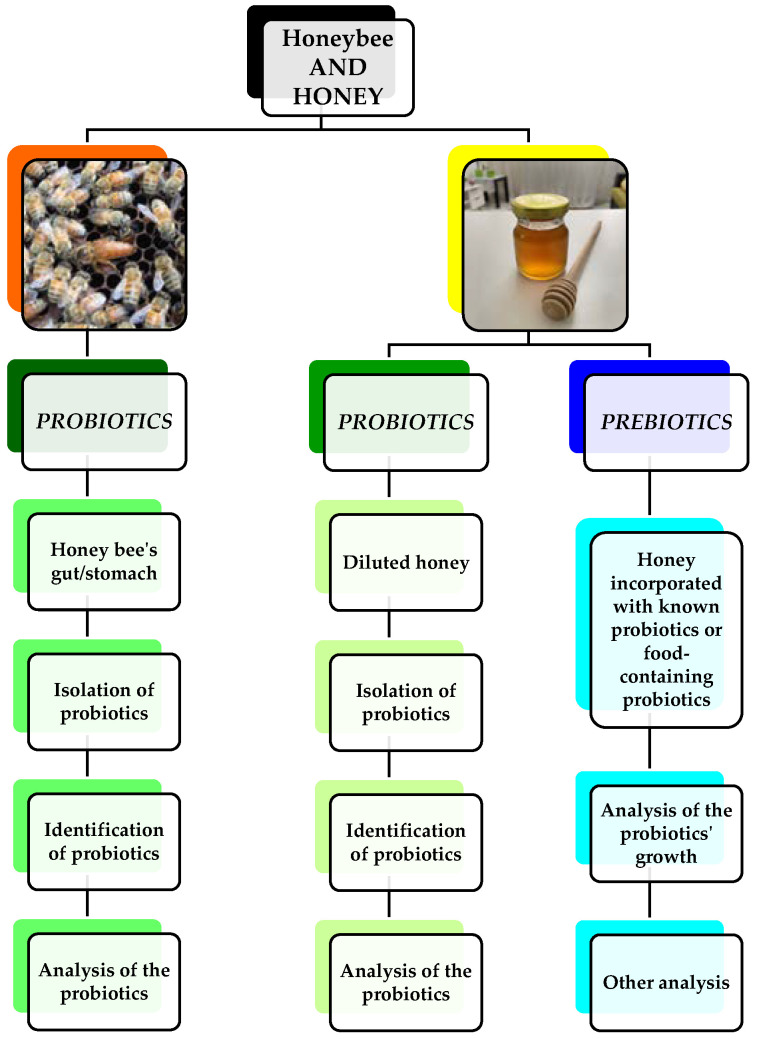
Flowchart of the honey bees and honey as a source of probiotics and prebiotics.

Since there are not many review articles that discuss these two topics, we find it necessary to highlight this subject due to its importance related to health issues. An overview of up-to-date information will increase and provide new knowledge regarding the topics. This article is based on original peer-reviewed articles, acquired from several online databases (PubMed, ScienceDirect, and Google Scholar) registered with the National Institutes of Health Malaysia Library. Some articles were retrieved through cross-reference searches of the review articles. Only full-length research articles published from January 2012 to December 2021 in the English language were considered. The article search was conducted using the keywords “honey”, “probiotic” and “prebiotic”, combined using Boolean operators (AND) adjacencies and truncations. Of the total number of articles obtained, only relevant and non-recurring articles were selected. Figure 1 shows the summarised process of isolating probiotics from honey bees and honey and utilizing prebiotics from honey.

## 2. Probiotic Properties of Honey Bees and Honey

In this article, only one of the honey bees’ products is featured; we chose honey since it is the most famous, widely consumed by the world’s population, and possessed many health benefits. Table 1 highlights the studies that have been performed in several countries on honey bees’ guts and honey as the origin of potential probiotics. The majority of honey bee probiotics have been identified from *A. mellifera* spp., with a few from the *A. cerana* spp., and *A. dorsata* spp. Probiotics isolated from the honey bee gut were composed of diverse microorganisms including *Bifidobacterium* and lactic acid bacteria (LAB), as well as fructophilic lactic acid bacteria (FLAB) which is a subgroup of LAB, yeasts, and other types of bacteria such as the *Bacillus* spp.

*Bifidobacterium* was less commonly discovered in the honey bees’ gut compared to the LAB. One study has shown the isolation of several *Bifidobacterium* species from the Japanese honey bee (*Apis cerana japonica*) gut. The isolates were shown to be strongly linked to bifidobacteria obtained from the European honey bees, implying that these bacteria are peculiar to the honey bee species. Furthermore, some of the bifidobacteria were capable of preventing the reproduction of *Melissococcus plutonius* which causes European foulbrood (EFB), indicating that they could be useful as probiotics in apiculture [22]. *Bacillus* species have also been the most isolated strains from the guts of healthy larvae and adult *Apis cerana* bees collected from Songkhram province, Thailand. However, the potential probiotic activity of those isolates has yet to be determined [23].

LAB is the most common probiotic isolated from the guts of honey bees, as shown in many studies conducted earlier (refer to Table 1). Various strains of LAB were harvested from the guts of *A. mellifera*, *A. cerana*, *A. dorsata*, and *A. florea* species. In most of the studies, the microorganisms were identified using the PCR technique with gene sequencing. *Lactobacillus* spp. was recovered from the digestive tract of *A. cerana indica* in various Karnataka locales [24]. Other *Lactobacillus* species such as *L. plantarum*, *L. pentosus*, and *L. fermentum* were prevalent within the gut of *Apis dorsata* with *L. plantarum* accounting for 51.02%. This study was the first to highlight the presence of *Lactobacillus* spp. in the Malaysian wild honey bees’ gut (*A. dorsata*). The quantity of LAB microbiota members discovered in the honey bee gut was found to be dependent on the season, origin, and volume of nectar [25]. Another species of *Lactobacillus*, known as *L. kunkeei* isolated from a healthy Yigilca honey bee gut, had shown immunity towards *M. plutonius* using the agar well diffusion technique. However, further experiments are needed to produce powerful pathogen-resistant probiotics so that bee treatments using antibiotics can be replaced or avoided [26]. Meanwhile, from the *A. mellifera* digestive tracts, lactobacilli species *L. plantarum* and *L. paraplantarum* were isolated, which exhibited antimicrobial activity toward harmful microorganisms that cause food deterioration and were found to degrade bile salts, preferring oxgall to taurodeoxycholate [27]. The same researchers also revealed that five *L. plantarum* species showed antioxidant activity, non-hemolytic properties, and antibiotic resistance. Analysis using Principal Component Analysis (PCA) highlighted two strains of *L. plantarum* (H28 and H24) to acquire the best properties tested, which can be designated as possible probiotic strain candidates [28]. In another study, two strains of *L. plantarum* (KX519413 and KX519414) harvested from the *A. cerana indica* gut showed survivability in the digestive tract environment, having very acidic pH due to the high content of bile salts and gastric juice. They are also hydrophobic, and able to produce biofilm and auto-aggregation, but at the same time, they can increase bacterial adherence and colonisation of the host digestive tract, which are important defense mechanisms against pathogens. The results implied that *L. plantarum* could be a promising probiotic candidate for food, pharmaceutical, and nutraceutical applications [29]. Different genera of LABs apart from the *Lactobacillus* spp. were also isolated from the stomachs and honey of *Apis* spp. which include *Lactococcus*, *Leuconostoc*, *Micrococcus*, *Streptococcus*, *Pediococcus*, and *Enterococcus*. LABs, as the prime symbiotic microorganism in bees, not only supply sufficient calories and nutrients but also a defense against infections [30]. In recent years, four of the five LABs isolated from the digestive system of Egyptian *A. mellifera* L., recognized as *E. faecalis* (MG890204, KX073783, EU594564), *L. brevis*, and *L. casei*, demonstrated antimicrobial action against pathogenic strains tested by producing acetic, oxalic, lactic, and glutaric acids [31].

FLAB strains were discovered alongside other LABs in an earlier study which comprises *F. fructosus* (two groups) and *L. kunkeei* (four groups). Antibacterial activity was observed in one strain of *L. kunkeei* against the pathogenic bacterium *M. plutonius*, due to the secretion of antimicrobial peptides or protein [32]. In another study, *Fructobacillus* (eight strains), *Lactobacillus* (seventy-two strains), *Enterococcus* (five strains), and one *Bifidobacterium asteroids* isolate were isolated from Georgian honey bees. In the existence of concanavalin, *Fructobacillus* sp. and *L. kunkeei* were capable of clumping up with the yeast cells and combining with the bacterial cells. The capacity to agglutinate and a mannose-specific adhesion shown by certain bacterial strains help to protect honey bees from unicellular microsporidian parasites. The cell membrane permeability of *L. kunkeei*, *F. tropaeoli*, and *F. pseudoficulneus* revealed a high adhesive potential (about 90%) trailed by *F. fructosus* and *Lactobacillus* spp. (about 80%), *L. casei* and *E. faecalis* (about 50%), and lastly *E. durans* (6%). The profoundly hydrophobic cell membrane interfaces of LAB as a potential probiotic act as an effective tool for them to adhere to and live in the host gut [33]. *F. fructosus* and *L. kunkeei* in *A. mellifera* guts, identified as heterofermentative LAB produce ethanol at trace level, as well as acetic and lactic acids from glucose. They also made it difficult for *Paenibacillus larvae* (prime honey bee pathogen) to thrive, suggesting that the tested LABs possessed good probiotic qualities [34]. The isolates displayed high tolerance survivability in the simulated intestinal tract conditions, showing good antibiotic susceptibility towards ampicillin, erythromycin, and tylosin, with hydrophobicity properties, able to enrich the development of biofilm and suppress the pathogenic microorganisms’ adherence to the membranes of the bee stomach. Furthermore, supplementing a sugar diet of *A. mellifera* with FLAB improves survival and substantially reduces mortality in the bees. The findings clearly show that *A. mellifera* FLAB symbionts have the potential to be used as probiotics [35].

All seven newly isolated species extracted from the intestines of *A. mellifera* composed of *F. fructosus*, *P. mirabilis*, *B. subtilis*, *B. licheniformis*, *L. kunkeei*, *E. kobei*, and *M. morganii* showed promising results for mortality testing on bee larvae that were infected by *P. larvae*. A normal diet incorporated with the gut bacteria significantly lowered the mortality percentage which occurred on day two and day three. The group treated with *L. kunkeei* exhibited the lowest mortality (56.67%) compared to the untreated group. Findings in this study suggested that probiotics from bees are capable of inhibiting pathogenic microorganisms, thus enhancing the survival rate of the larvae [36]. One species of fructophilic, facultative anaerobic gram-positive, rod-shaped bacterium, known as *A. kunkeei* was also harvested from *A. mellifera* gut. It produces several valuable enzymes, shows low tolerance for antibiotics, prevents the growth of *P. aeruginosa*, and can survive in a high sugar solution at a low temperature for one month. These features suggested that *A. kunkeei* can be used for probiotic action in fruit preparation for hospitalized patients with a weak immune system. The survival rate of this strain in a gut model was similar to *Lacticaseibacillus rhamnosus* GG, a control probiotic used in the study [37].

Probiotics from the genus *Bacillus* were also known to inhabit the honey bee’s gut and have been secluded from the Japanese honey bee. One isolate inhibits *M. plutonius* while the *A. mellifera* larvae were still surviving at the end of the fifth day, indicating a considerably lower mortality rate than the untreated group [38]. A pair’s whole genomic sequence of newly isolated *B. subtilis* strains (MENO2 and HMNig-2) from the bee gut microbiome and honey, respectively, reported probiotic characteristics and produced many valuable components, including levan [39]. Levan has been utilized as a flavor carrier, food coating substance, stabilizing, emulsifying, encapsulating, thickening, and surface-finishing agents in the food industry. It has also shown potential as an antiviral, anti-inflammatory, hyperglycemic inhibitor, and antihyperlipidemic agent in the biomedical field [51]. Several other types of *Bacillus* sp. and yeasts were also isolated from the gut of *A. mellifera* species such as *B. licheniformis*, *P. polymyxa* (*B. polymyxa*), *W. anomalus*, *L. thermotolerans*, and *Z. mellis*. They exhibited a high potentiality as probiotics by showing tolerance to a very low pH (up to 1.5) and being able to survive in a high concentration of bile salt (up to 3%) after 3 h of incubation. Antimicrobial activities were reported with the presence of several compounds such as heptadecane, palmitic acid, dodemorph, paraldehyde, octadecanoic and fenoprofen [40].

Honey is also known to accommodate probiotic organisms coming from the honey bee’s gut at the time of honey-making activities. LAB isolated from *A. dorsata* honeycomb is comprised of *L. kunkeei* and other *Lactobacillus* sp. This is the first published investigation of lactobacilli in honeycombs of the huge Malaysian wild honey bee *A. dorsata*, showing honeycombs to function as potential sources of novel probiotic microorganisms as natural food preservatives [41]. *Leuconostoc* sp. (*L. mesenteroides*), one of the strains in the LAB group that was harvested from *A. mellifera* honey, exhibited resilience towards intestinal tract stressors, and hydrophobic nature as well as antimicrobial activities in opposition to *Staphylococcus aureus* and *Escherichia coli* [42]. Other types of LABs obtained from *A. mellifera* honey were sensitive toward *L. monocytogenes* and *E. coli* with higher sensitivity towards *E. coli*. The antibacterial action of the LAB is shown by disintegrating the outer part of the bacterial cell wall and reducing the surrounding pH to prevent pathogens from proliferating or surviving longer [43].

There have also been findings of the presence of *Bacillus* sp., in honey. Earlier in 2012, Esawy and colleagues [44] isolated *Bacillus* spp. from spores found in honey from three Gulf countries (Libya, Saudi Arabia, and Egypt). They exhibited good tolerance to pH 3 and pH 9 for up to 6 h with varying degrees of viability, resistance to 0.3% bile and pancreatic enzyme (except for one isolate), and negative results for hemolytic testing. The isolates also demonstrated antioxidant and antimicrobial properties, suggesting that they could flourish in the intestinal tract and function as potential antibiotic producers [44]. The *Bacillus* and *Lactobacillus* species (*B. subtilis*, *B. brevis*, *B. megaterium*, and *L. acidophilus*) isolated from the Iranian honey demonstrated various antibacterial activities, inhibiting several pathogens and showing the absence of antibiotic resistance for most of the isolates. These parameters together with high resistance to acidic pH and concentrated bile salts are considered favorable characteristics of microorganisms used in the manufacturing of food probiotics. During the evaluation period, all strains conferred resistance to 1% bile salt, and *B. megaterium* were found to possess outstanding resistance to relatively low pH for 4 h which was beyond the normal time transition of food in the stomach which is between 2 to 3 h. *B. subtilis* and *B. megaterium*, which performed better compared to *Lactobacillus* sp., were found to be significant probiotic candidates since no virulence genes were detected in them, thus may be deemed secure for future implementation [45]. *B. subtilis* was also isolated from different types of honey such as the mountain and persimmon honey, along with another species *B. endophyticus* [46]. The *B. subtilis* was more tolerant to acidity at pH 2 in comparison to *B. endophyticus* after 24 h of treatment. However, at higher pH of 2.5–3.0, *B. endophyticus* was more tolerant compared to *B. subtilis*. Furthermore, the isolates were able to withstand various alkaline pH and make adjustments to a very high alkaline environment (pH 10) for a 24 h duration. Among the important probiotic requirements are the resilience features to extreme acidity and the ability to thrive in transit since the pH of the stomach can be as low as 1.5–2.0 [52]. Bile salt resistance analyses are required to evaluate the capacity of the isolates to survive in the gastrointestinal tract, examples are the resistance to 0.3 and 0.7% bile salt. The absence of the hemolytic enterotoxin gene and the positive susceptibility to antibiotics showed that certain safety criteria for future application have been met, particularly in the food and pharmaceutical industries [46]. One *Bacillus* sp. isolated from honey was found to inhibit pathogenic fungus, *Candida albicans*, and bacteria *E. coli* and *S. aureus*. Upon the attachment of the *Bacillus* sp., the pathogens’ shape changes and is damaged, causing disruption and leakage on their cell wall. As an effective probiotic, it may help to control the host’s microbiota [47]. Raw honey was found to harbour other types of *Bacillus* sp. apart from *B. subtilis*, such as *B. mycoides*, *B. thuringiensis*, *B. amyloliquefaciens*, and *B. velezensis* identified using PCR and sequences analysis [48].

*A. cerana indica* honey becomes a carrier for *Gluconobacter oxydans*, a gram-negative bacterium from the *Acetobacteraceae* family which exhibits a probiotic quality [49]. *G. oxydans* isolated from freshly harvested honey was found to possess siderophorogenic potential and non-hemolytic activity which assure security as a possible probiotic microorganism. The probiotic’s capability to persist in the adverse conditions of the human gastrointestinal tract is revealed by its acid and bile tolerance characteristics of which the *G. oxydans* manage to endure a 2% bile salt concentration. The percentage of isolates that are auto-aggregated was found to be linearly related to incubation time, with notable % adherence to xylene being more than to ethyl acetate and chloroform. The link involving mucous membrane adherence and auto-aggregation capacity was previously described by Del Re et al. (2000) [53]. The current study revealed that *G. oxydans* developed a trihydroxamate siderophore that helps promote bacterial growth suggesting a possible probiotic which can offer health advantages to the host [49].

Apart from bacteria, yeast strains were also discovered in honey. Recently, one hundred and six yeast strains were isolated from three honey bee samples, with more than half of the isolates demonstrating an acceptable survival rate under restored gastrointestinal circumstances; pH ranged from 2.0–2.5, bile salt of 0.3% (*w*/*v*), and temperature was 37 °C. Some of the isolated strains appeared to express elevated auto-aggregation of 80 to 100% and might lead to the use of numerous types of sugars, including xylose and galactose. *S. cerevisiae* species were found to generate a high magnitude of antimicrobial acids derived from the sugar industry. Moreover, *M. guilliermondiii* produced 0.49 g of polyol xylitol per gram of consumed xylose. The yeast isolates may provide potential probiotics for the functional food and feed industry or metabolite producers for the lignocellulosic biorefinery [50].

## 3. Prebiotic Properties of Honey

Earlier research indicated that the prebiotic properties of honey are mainly due to the oligosaccharides and low molecular weight polysaccharides attached by the β-glycosidic linkages [20]. Prebiotics are hydrolyzed by the native intestinal microflora as human digestive enzymes do not possess β-glycosidases. Inulin, fructose-oligosaccharides (FOS), pyrodextrins, lactulose, and xylooligosaccharide are among the well-known prebiotics [54]. Several investigations into the possible prebiotic characteristics of honey have been undertaken in various regions of the world. Table 2 summarizes the different types of honey as a source of prebiotics and their effects on probiotics commonly found in the human intestinal system. Table 3 shows the efficiency of honey as a prebiotic for probiotics in other foods such as dairy and non-dairy products.

Many types of honey were investigated for their prebiotic properties on LAB and *Bifidobacterium*. The probiotic ability to co-aggregate with the pathogens increased with the addition of honey, while the self-digestion reaction was greatly reduced when inulin was present. Co-aggregation capabilities allow probiotics to create a barrier that stops colonization by pathogens. Cell surface hydrophobicity is the probiotics’ capacity to attach to the gut endothelium. Inulin significantly increased cell membrane hydrophobicity in comparison to honey. Autolysis occurs when bacteria cell naturally deteriorates due to maturity or adverse biological factors, causing the autolysin enzymes to hydrolyse the cell wall peptidoglycan. From this study, it was found that *L. acidophilus* showed better attributes in prebiotics inulin than honey [55]. The viable count of *L. acidophilus* and *B. bifidum* were much higher when cultured in carbohydrate-free MRS broth fortified with sesame honey compared to the unfortified broth. At the same time, sesame honey also promotes antibacterial activity against several pathogens such as *E. coli*, *V. cholerae*, *S. typhi*, and *S. typhimurium* with the lowest minimum inhibitory concentration (12.5%) toward *S. typhi* and *S. typhimurium* [56]. Chestnut honey was discovered to promote the flourishing of *L. acidophilus* LA-5 and *L. rhamnosus* GG, displaying probiotic features such as autoaggregation and surface hydrophobicity. Furthermore, as compared to probiotics or honey alone, probiotics grown with honey were more cytotoxic to cancer cell types [57]. Wild polyfloral honey from Cameroon was observed to thrive and sustain survivability of *L. plantarum* 29 V, persisting for up to 28 days without compromising the quality of honey. In vivo studies on hypercholesterolemic rats showed that treatment using the probiotic in honey, significantly reduced total cholesterol, LDL-cholesterol, and lipids in the serum of rats. Moreover, HDL-cholesterol was elevated and the atherosclerotic production was remarkably reduced [58]. Seven types of Italian monofloral honey showed different capabilities of inhibiting pathogens including *Acinetobacter baumannii*, *E. coli*, *Listeria monocytogenes*, *Pseudomonas aeruginosa*, and *Staphylococcus aureus*. The honey was capable of inhibiting the production of pathogen biofilms, suppressing adhesion activity, and reacting to full-grown biofilm. However, on the other hand, it did not influence *E. Coli* mature biofilm inhibition or metabolism. The LAB probiotics which comprise *L. acidophilus*, *L. gasseri*, *L. casei*, *L. rhamnosus*, and *L. plantarum* were shown to grow faster in honey with prebiotic potential [59].

The growth of bifidobacterial species isolated from the faeces of breastfed infants with medical-grade honey and inulin was enhanced with the non-substantial difference among both. Inulin (0.4%) and honey (3%), exert maximum prebiotic activity on *B. longum*, *B. breve*, and *B. bifidum* [60]. A prospective randomized control trial using sterilized, unprocessed clover honey to treat prematurely born infants revealed the effectiveness of honey in increasing the number of *B. bifidum* in infants treated with different concentrations of honey (5 g, 10 g, and 15 g). Similarly, an increased number of lactobacilli was observed with 10 g of honey. RT-PCR results confirmed the presence and escalating number of *B. bifidum* and lactobacilli in infants given 15 g of honey. Apart from that, *Enterobacter* colonization was found to be significantly decreased in the stools of infants given 5 g and 10 g of honey milk solution [61]. Previously, Buckwheat honey, containing carbohydrates and phenolic compounds was shown to increase the growth of Bifidobacteria from the human gut and suppress the growth of pathogenic microbes such as *Prevotella*, *Faecalibacterium*, and *Lachnospiraceae incertae sedis* compared to the control group [62]. Manuka honey oligosaccharides substantially decreased the adherence of *E. coli*, *Staphylococcus aureus*, and *Pseudomonas aeruginosa* to HT-29 cells by 40%, 30%, and 52%, respectively. These substances could bind to bacterial and/or epithelium membrane receptors reducing the possibility of infection and eliminating the risk of bacterial colonisation [63]. The prebiotic attribute of Jarrah honey on gut microbiota in mice resulted in a significant increase in faecal moisture level and relieving constipation due to the alteration of microbial ecology. The microbial diversity was largely enhanced on day 12 due to the application of honey, producing healthier gut ecosystems of the host [64]. Recently, melezitose and the carbohydrate profile of the Giant Willow Aphid (*Tuberolachnus salignus*) honeydew honey were identified [65]. Melezitose was the biggest component of oligosaccharides, comprising 27.4% of 38.4% of the total content of oligosaccharides. The hydrolysis of melezitose with acid produces glucose and fructose monomers, which mimic the process of hydrolysis by digestive enzymes in the stomach. Results showed that melezitose is not affected by acid hydrochloric or enzyme (α-glucosidase) hydrolysis under similar conditions to human digestion and could be a potential prebiotic source [65]. In 2021, a study using AMF™ Manuka honey showed that a higher number of *L. reuteri* were observed when grown in the Manuka honey with the highest Unit Manuka Factor (UMF) of 20^+^. However, when compared to the lower level of UMF (5, 10, and 15), the outcomes were not statistically significant [66].

Honey is well explored in dairy and non-dairy foods as a prebiotic source for probiotics (Table 3). Both types of honey, either monofloral or polyfloral, can serve as good prebiotic sources in foods. Honey is combined with dairy products derived from the fresh milk of cows [68,71,72,78,79], goats [75,77], camels [71], and buffalos [73] to produce yogurt. Some researchers also utilized skimmed milk powder to produce yogurt [69,70,76]. As for the non-dairy products, kefir [74], soy milk [67,80], and hydrolyzed soybean extract [74] were chosen to replace the animal’s milk. The most common starter culture probiotics used to produce yogurt are the *S. thermophilus* and *L. delbrueckii* ssp. *bulgaricus*. The findings of numerous investigations revealed that the number of probiotics in honey-containing foods is significantly enhanced. Monofloral honey (chestnut, acacia, lime honey) and polyfloral honey (eucalyptus, greenbrier) in yogurt were found to be good prebiotic sources for cultivating Bifidobacteria strains of diverse subspecies [67,69]. Saudi Arabian raw honey [68], black locust honey [71], Kerala natural honey [72], African commercial honey [76], marjoram honey [77], and pine honey [78] were all found to be suitable for cultivating *Lactobacillus* and *Bifidobacterium* species in the dairy and non-dairy goods. It was revealed in 2020 that Manuka honey of the brand AMF™ 15+ fortified in yogurt significantly increased the viable count of *L. reuteri* surpassing the recommended value of 7 log CFU/mL compared to the other brands tested (Manuka Blend and UMF™ 18+) [79]. A clinical trial in postmenopausal women treated with *L. plantarum*-fermented soymilk-honey for 90 days disclosed a notable reduction in the levels of osteocalcin in the participants’ blood serum, implying that honey as a source of prebiotics can assist probiotics in surviving longer for treatment purposes [80].

## 4. Conclusions

Based on the results, not too many experimental studies have been conducted on the probiotic and prebiotic aspects of honey bees and honey within the last ten years. Studies on honey bee probiotics are mainly performed on certain species only such as the *A. mellifera*, *A. dorsata*, and *A. cerana*. Further investigations are needed to produce safe probiotics with high resistance to pathogenic microorganisms for animal and human consumption or disease treatment. A similar situation was also seen in prebiotic studies, where most of the research was mainly conducted in-vitro using the normal microflora inhabiting the human intestine from fecal samples that were treated with various concentrations of the selected honey. In conclusion, honey bees and honey, which have the potential to be good sources of probiotics and prebiotics, need to be given greater attention and more in-depth research so that they can be taken to the next level. Further research will increase the likelihood of developing an alternative therapeutic for antibiotic-resistant pathogens.

## Figures and Tables

**Table 1:** Potential probiotics in the bees’ gut and honey.

Probiotic	Source	Origin/Country	Reference
*Bifidobacterium* spp.	*Apis cerana japonica* gut	Tsukuba, Japan	[22]
*Bifidobacterium* spp., *Lactobacillus* spp., *Bacillus* spp.	*Apis cerana indica* gut	Samut-Songkhram, and Chumphon, Thailand	[23]
*Lactobacillus* spp.	*Apis cerana indica* gut	Karnataka, India	[24]
*Lactobacillus plantarum*, *Lactobacillus pentosus*, *Lactobacillus fermentum*	*Apis dorsata* gut	Terengganu, Malaysia	[25]
*Lactobacillus kunkeei* strains	Yigilca honey bee gut	Duzce, Turkey	[26]
*Lactobacillus plantarum*, *Lactobacillus paraplantarum*, *Lactobacillus plantarum* strains	*Apis mellifera* gut	Menoua, Cameroon	[27] [28]
*Lactobacillus plantarum* strains	*Apis cerana indica* gut	Kerala, India	[29]
Lactic Acid Bacteria (LAB) genera: *Enterococcus*, *Lactobacillus*, *Micrococcus*, *Lactococcus*, *Streptococcus*, *Pediococcus*, *Leconostoc*	*Apis cerana indica* Fabricius, *Apis mellifera* Linnaeus, *Apis florea* Fabricius, & *Apis dorsata* Fabricius guts and honey	Tamil Nadu, India	[30]
*Enterococcus faecalis* strains, *Lactobacillus brevis*, *Lactobacillus casei*	*Apis mellifera* gut	Cairo, Egypt	[31]
*Fructobacillus fructossus* strains, *Lactobacillus kunkeei* strains	*Apis mellifera mellifera*, *Apis mellifera ligustica* and hybridized bee guts, larvae and honey	Aland Island, Finland	[32]
*Lactobacillus kunkeei* strains (sixty-six strains), *Lactobacillus casei* (one strain), *Lactobacillus* spp. (five unidentified strains), *Fructobacillus fructosus* strains (eight strains), *Enterococcus* (five strains), *Bifidobacterium asteroids*	*Apis mellifera* gut	The Caucasus Mountains, and Kolkheti Valley, Georgia	[33]
*Lactobacillus kunkeei* strains, *Lactobacillus fructosus* strains	*Apis mellifera* gut	Lublin, Poland	[34]
*Lactobacillus kunkeei* strains, *Fructobacillus fructossus* strains	*Apis mellifera* Linnaeus gut	Pulawy, Poland	[35]
*Fructobacillus fructossus*, *Proteus mirabilis*, *Bacillus subtilis*, *Bacillus licheniformis*, *Lactobacillus kunkeei*, *Enterobacter kobei*, *Morganella morganii*	*Apis mellifera jemenitica* gut	Riyadh, Saudi Arabia	[36]
*Apilactobacillus kunkeei* strains	*Apis mellifera* Linnaeus gut	N/A	[37]
*Bacillus* spp.	*Apis cerana japonica* gut	Tsukuba, Japan	[38]
*Bacillus subtilis* strains	Honey bee gut and honey	N/A	[39]
*Bacillus licheniformis*, *Paenibacillus polymyxa* (*Bacillus polymyxa*), *Wickerhamomyces anomalus*, *Lachancea thermotolerans*, *Zygosaccharomyces mellis*,	*Apis mellifera carnica* gut *Apis mellifera ligustica* gut	Giza, Egypt	[40]
*Lactobacillus kunkeei* strains, *Lactobacillus* spp.	Honey (*Apis dorsata*)	Kedah, Malaysia	[41]
*Leuconostoc mesenteroides* strains	Honey (*Apis mellifera*)	Algeria	[42]
Lactic Acid Bacteria (Species and subspecies not mentioned)	Honey (*Apis mellifera*)	Indonesia	[43]
*Bacillus* spp.	Commercial honey (Libya, Saudi Arabia and Egypt)	N/A	[44]
*Bacillus subtilis*, *Brevibacillus brevis*, *Bacillus megaterium* strains, *Lactobacillus acidophilus*	Local honey	Iran	[45]
*Bacillus subtilis* strains *Bacillus endophyticus*	Mountain honey Persimmon honey (commercial)	Nigeria Egypt	[46]
*Bacillus* spp.	Honey	China	[47]
*Bacillus subtilis*, *Bacillus mycoides*, *Bacillus thuringiensis*, *Bacillus amyloliquefaciens*, *Bacillus velezensis*	Raw honey (Polyfloral)	Romania	[48]
*Gluconobacter oxydans*	Honey (*Apis cerana indica*)	Tamil Nadu, India	[49]
*Saccharomyces cerevisiae* strains, *Meyerozyma guilliermondiii*	Raw honey (*Apis dorsata fabricius*)	Ratchaburi, Thailand	[50]

N/A = not available.

**Table 2:** Prebiotic potential of honey.

Probiotic	Sources of Prebiotic	Country	Key Findings	Reference
*Lactobacillusacidophilus* strains	Honey	India	- Honey enhanced the coaggregation of *E. coli* with *L. acidophilus* NCDC 291 more than with *L. acidophilus* NCDC 13. - Both strains showed a higher capability of autoaggregation and hydrophobicity, and reduced autolytic activity with inulin compared to honey.	[55]
*Lactobacillus acidophilus*, *Bifidobacterium bifidum*	Sesame honey (*Sesamum indicum*)	India	- Sesame honey (5%) exhibited selective and significant growth-supporting properties of the probiotics.	[56]
*Lactobacillus acidophilus*, *Lactobacillus rhamnosus*	Chestnut honey	Turkey	- Chestnut honey has positively impacted probiotic bacteria by increasing growth and modulating probiotic properties such as auto-aggregation and surface hydrophobia.	[57]
*Lactobacillus plantarum* strain	Wild honey (Polyfloral)	Cameroon	- *L. plantarum* 29 V can survive for 28 days at 4 °C and 25 °C due to their ability to resist lower pH and the presence of oligosaccharides (fructo- and gluco-oligosaccharides) in honey recognized as prebiotics. - Hypercholesterolemic rats treated with honey containing *L. plantarum* 29 V showed an increase in HDL-cholesterol level and lowers total cholesterol, LDL-cholesterol, triglycerides and atherosclerosis index in serum.	[58]
*Lactobacillus acidophilus*, *Lactobacillus gasseri*, *Lacticaseibacillus casei*, *Lacticaseibacillus rhamnosus*, *Lactiplantibacillus plantarum*	Fir, strawberry tree, ivy, tree of heaven, sulla, cardoon, rhododendron honey (Commercial, organic, monofloral honey)	Italy	- Fir, ivy, and sulla honey (1% and 2%) stimulate the growth of all the probiotics tested with various actions compared to more specific cardoon honey.	[59]
*Bifidobacterium longum* strains, *Bifidobacterium breve*, *Bifidobacterium bifidum*	Agmark grade honey	India	- Honey showed a prebiotic effect on all isolates, especially on *B. longum* at 3% and 5% honey.	[60]
*Bifidobacterium bifidum* and Lactobacilli	Clover honey (Unprocessed and sterilised)	Egypt	- Increased *B. bifidum* colony counts were observed in all honey-supplied group (Group A-5 g, B-10 g, and C-15 g honey), with group B, showing a significant rise in comparison with the control.	[61]
Bifidobacteria	Buckwheat honey	China	- Buckwheat honey assists in propagating native Bifidobacteria and prohibits the growth of the pathogenic bacterium in the gut system.	[62]
N/A	Manuka honey (MGO™)	Ireland	- Honey-containing oligosaccharides inhibited *P. aeruginosa* (52%), *E. coli* O157:H7 (40%) and *S. aureus* (30%) in the cancer cells.	[63]
Microbiota of the mice gut	Jarrah honey	China	- Honey helps to retain more water in the faecal and relieves constipation and suppresses the growth of *Desulfovibri*.	[64]
N/A	Giant Willow Aphid honeydew honey (*Tuberolachnus salignus*)	New Zealand	- A high concentration of melezitose can act as a prebiotic for the human digestive system since it is not hydrolysed by acid and is only partially hydrolysed by α-glucosidase.	[65]
*Limosilactobacillus reuteri*	Manuka honey (Drapac DrKiwi AMF5, AMF10, AMF15 and AMF20)	New Zealand	- High sugar and oligosaccharides contributed to higher probiotic cell biomass of AMF20, but no obvious pattern in biomass with a decrease in AMF concentration.	[66]

N/A = not available.

**Table 3:** The prebiotic potential of honey incorporated into other foods.

Probiotic	Sources of Prebiotic	Country	Key Findings	Reference
*Bifidobacterium lactis*, *Bifidobacterium longum*	Chestnut and Acacia honey (Monofloral) in fermented soymilk	Croatia	- The addition of honey at 5% and 10%, increases the number of bifidobacteria, lowers the pH, and decreases raffinose and stachyose content, making them an effective prebiotic.	[67]
*Lactobacillus delbreuckii* ssp. *bulgaricus*, *Streptococcus thermophilus*, *Bifidobacterium bifidum*, *Lactobacillus rhamnosus*, *Lactobacillus reuteri*	Raw honey in fermented cow’s milk	Saudi Arabia	- Honey (3%) is beneficial in increasing all probiotic strains population with no significant variation in organoleptic scores compared to inulin.	[68]
*Lactobacillus delbreuckii* ssp. *bulgaricus*, *Streptococcus thermophilus*, *Bifidobacterium breve* strains, *Bifidobacterium longum* strains,	Eucalyptus and Greenbrier honey (Polyfloral) or Lime honey (Monofloral) in yogurt (skimmed milk)	Algeria France	- Honey used at 5 or 10% (*w*/*v*) was stimulatory for Bifidobacterial growth, however, 5% honey seems to be the best for commercial production containing Bifidobacteria since it improves fermentation prowess and stable shelf life.	[69]
*Lactobacillus delbrueckii* ssp. *bulgaricus*, *Streptococcus salivarius* ssp. *thermophilus*	Honey in yogurt (skimmed milk)	Pakistan	- The addition of 10% honey to yogurt resulted in a higher viable count for *L. delbrueckii* ssp. *bulgaricus*, and *S. thermophilus* compared to 5%, 15% and 20% honey.	[70]
*Lactobacillus acidophilus*, *Bifidobacterium animalis* ssp. *lactis*, *Streptococcus thermophilus*	Black locust honey (*Robinia pseudoacacia* L.) in yogurt (camel and cow’s milk)	Hungary UAE	- Yogurt, supplemented with 5% honey, prolonged the survival of *B. animalis* ssp. *lactis* in yogurt (camel’s milk) stored in a refrigerator for up to 5 weeks.	[71]
Lactic acid bacteria (LAB)	Natural Kerala honey, Honey oligosaccharides in fermented milk (cow’s milk)	India	- Honey is the most effective prebiotic in enhancing the growth and functionality of LAB by maintaining the viability, pH, and titrable acidity of the fermented milk.	[72]
*Lactobacillus delbrueckii* ssp. *bulgaricus*, *Streptococcus thermophilus*, *Lactobacillus acidophilus*, *Bifidobacterium bifidum*	Sedr honey in yogurt (buffalo’s milk)	Egypt	- Yogurt with 10% honey, increased the growth of *L. delbrueckii* ssp. *bulgaricus*, and *S. thermophilus* compared to using 5%, 15%, and 20% honey.	[73]
*Lactobacillus statsumensis*, *Leuconostoc mesenteroides*, *Bacillus megaterium*, *Saccharomyces cerevisiae*, *Lachancea fermentati*	- Local honey in Mexican kefir beverage - Tibetan kefir beverage (hydrolyzed soybean extract, bovine colostrum and cow’s milk)	Brazil	- Honey-kefir beverage produced many potential probiotic bacteria and yeasts. - It also showed lower content of lactose, protective against DNA damage, and high antioxidant activity and sensory quality.	[74]
*Streptococcus thermophiles*, *Lactobacillus acidophilus*, *Bifidobacterium* sp.	Local commercial honey in yogurt (goat’s milk) and tamr (*P. dactylifera* L.)	Egypt	- Honey supplementation at different concentrations (1%, 2%, and 3%) had a great influence on activity and the cell count of probiotics., thus making it an effective functional prebiotic.	[75]
*Lactobacillus* *acidophilus*, *Bifidobacterium* sp.	African commercial honey in yogurt (skimmed milk powder)	N/A	- Honey at 5 and 10% concentrations interacted with storage times (35 days under refrigeration (2–4 °C), significantly affecting cell counts, and remained viable showing good prebiotic attributes.	[76]
*Lactobacillus casei*	Marjoram honey in yogurt (goat’s milk)	Egypt	- Marjoram honey in yogurt exhibited an increased colony count of *L. casei* and positively affected its organoleptic properties which led to an increase in the period of vitality for the probiotic.	[77]
*Lactobacillus delbrueckii* ssp. *bulgaricus*, *Streptococcus thermophilus*, *Lactobacillus rhamnosus*, *Lactobacillus acidophilus*, *Lactobacillus plantarum*, *Bifidobacterium animals* ssp. *lactis*	Pine honey in yogurt (cow’s milk)	Turkey	- The number of *S. thermophilus* is higher compared to *L. delbrueckii* and *L. acidophilus* when added with honey (2%, 4%, and 6%).	[78]
*Lactobacillus delbrueckii* ssp. *bulgaricus*, *Streptococcus thermophilus*, *Lactobacillus reuteri*	Manuka honey (Blend, UMF™ 18+, AMF™ 15+ and AMF™ 20+) in yogurt (cow’s milk)	New Zealand	- AMF™ 15+ manuka honey in yogurt significantly increased the number of probiotics exceeding the recommended level (7 log CFU/mL) compared to the other honey types.	[79]
*Lactobacillus casei* ssp. *casei*, *Lactobacillus plantarum*	Honey in fermented soymilk	Indonesia Poland	- *L. plantarum*-fermented soymilk-honey therapy for 90 days effectively reduced osteocalcin content in blood serum.	[80]

N/A = not available.

## Data Availability

Not applicable.
